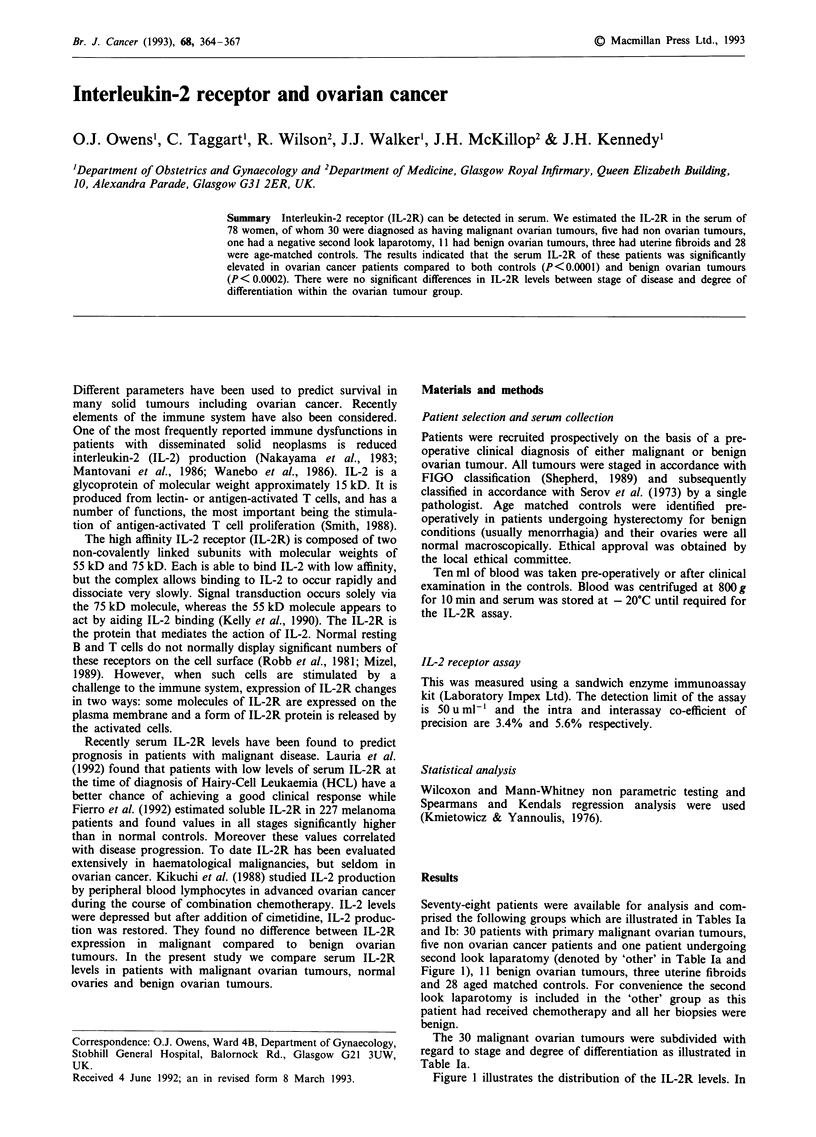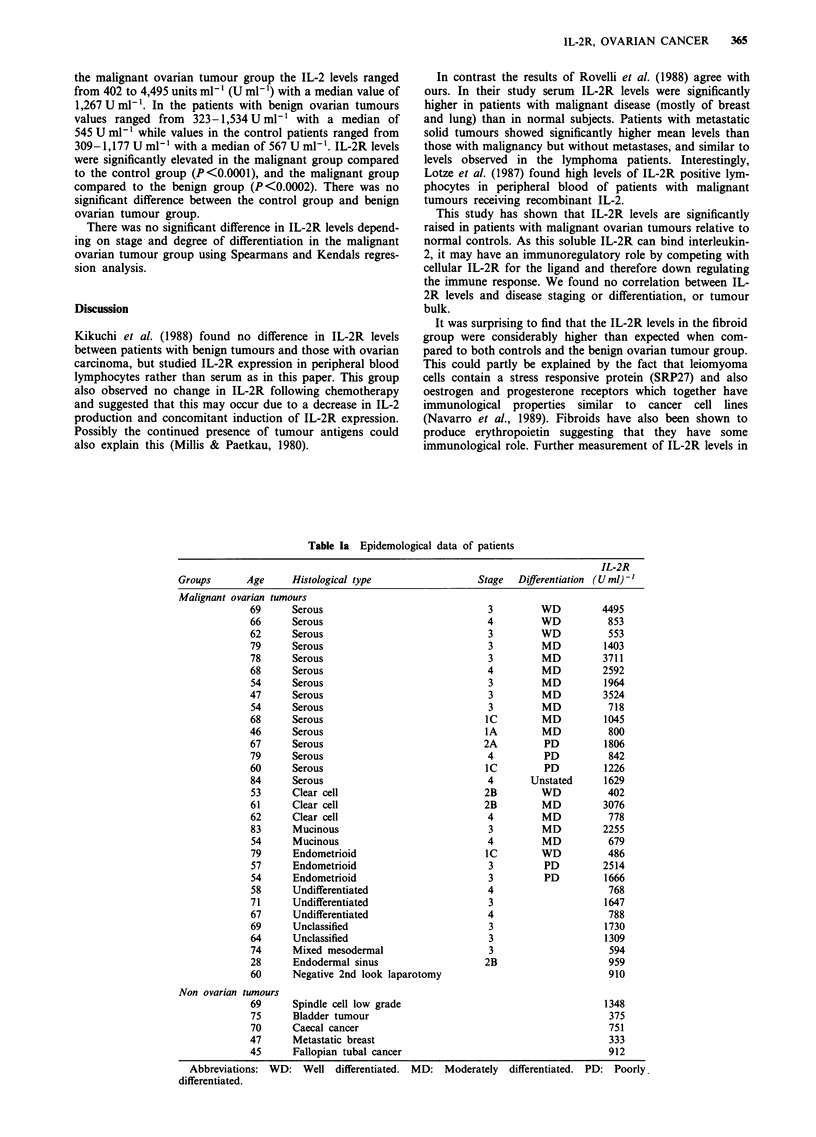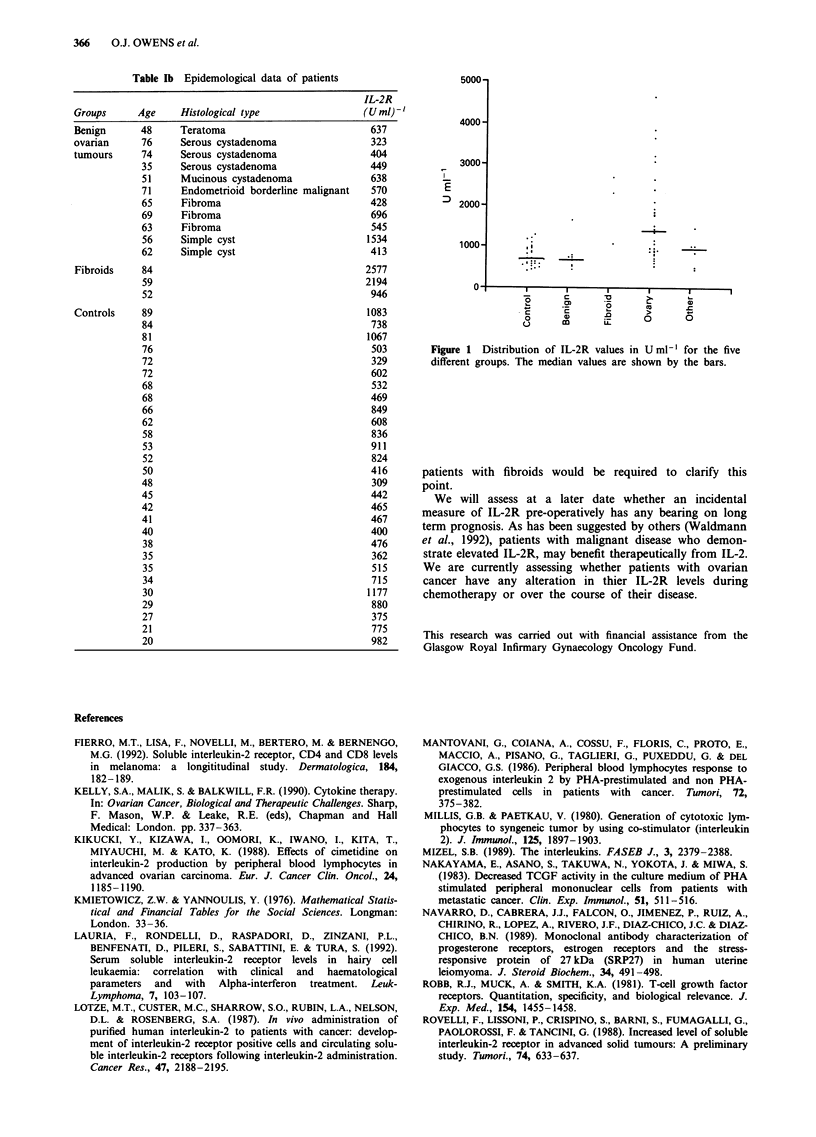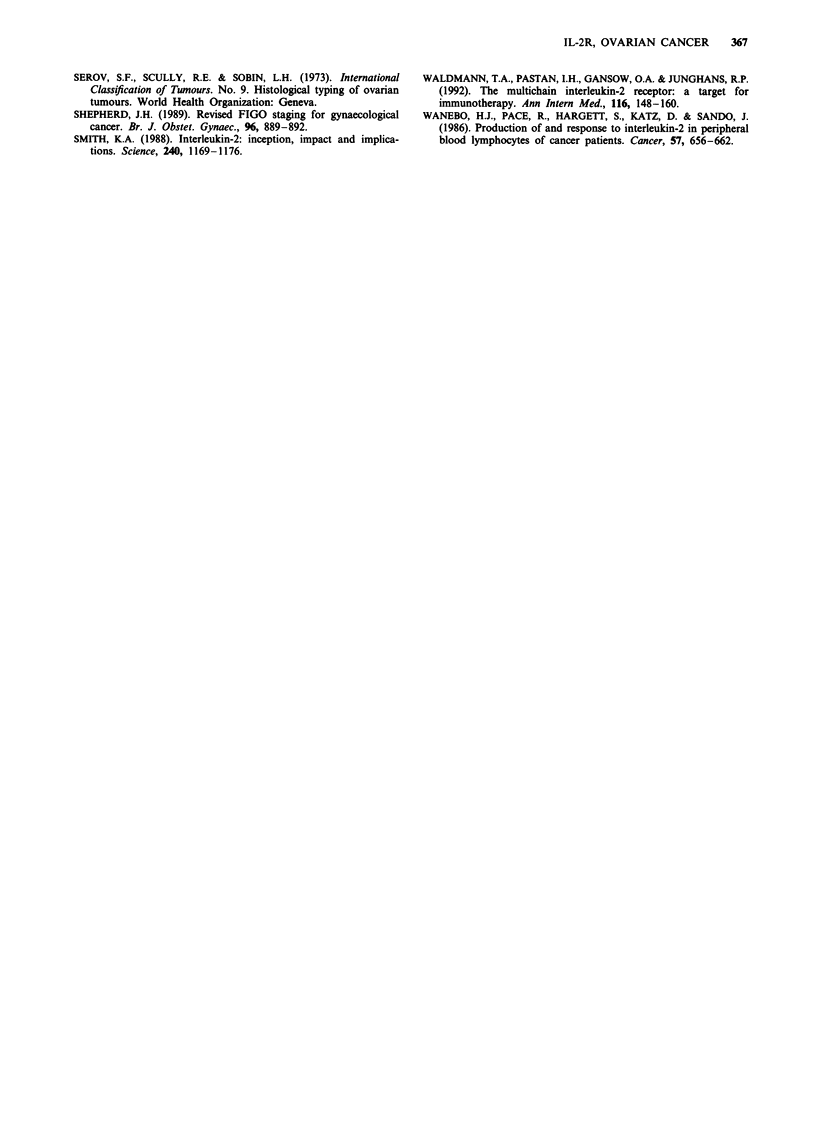# Interleukin-2 receptor and ovarian cancer.

**DOI:** 10.1038/bjc.1993.341

**Published:** 1993-08

**Authors:** O. J. Owens, C. Taggart, R. Wilson, J. J. Walker, J. H. McKillop, J. H. Kennedy

**Affiliations:** Department of Obstetrics and Gynaecology, Glasgow Royal Infirmary, UK.

## Abstract

Interleukin-2 receptor (IL-2R) can be detected in serum. We estimated the IL-2R in the serum of 78 women, of whom 30 were diagnosed as having malignant ovarian tumours, five had non ovarian tumours, one had a negative second look laparotomy, 11 had benign ovarian tumours, three had uterine fibroids and 28 were age-matched controls. The results indicated that the serum IL-2R of these patients was significantly elevated in ovarian cancer patients compared to both controls (P < 0.0001) and benign ovarian tumours (P < 0.0002). There were no significant differences in IL-2R levels between stage of disease and degree of differentiation within the ovarian tumour group.


Br. J. Cancer (1993), 68, 364–367 © Macmillan Press Ltd., 1993

### Interleukin-2 receptor and ovarian cancer

O.J. Owens[1], C. Taggart[1], R. Wilson[2], J.J. Walker[1], J.H. McKillop[2] & J.H. Kennedy[1]

[1]*Department of Obstetrics and Gynaecology and* [2]*Department of Medicine, Glasgow Royal Infirmary, Queen Elizabeth Building, 10, Alexandra Parade, Glasgow G31 2ER, UK.*

**Summary** Interleukin-2 receptor (IL-2R) can be detected in serum. We estimated the IL-2R in the serum of 78 women, of whom 30 were diagnosed as having malignant ovarian tumours, five had non ovarian tumours, one had a negative second look laparotomy, 11 had benign ovarian tumours, three had uterine fibroids and 28 were age-matched controls. The results indicated that the serum IL-2R of these patients was significantly elevated in ovarian cancer patients compared to both controls ($P<0.0001$) and benign ovarian tumours ($P<0.0002$). There were no significant differences in IL-2R levels between stage of disease and degree of differentiation within the ovarian tumour group.

Different parameters have been used to predict survival in many solid tumours including ovarian cancer. Recently elements of the immune system have also been considered. One of the most frequently reported immune dysfunctions in patients with disseminated solid neoplasms is reduced interleukin-2 (IL-2) production (Nakayama *et al.*, 1983; Mantovani *et al.*, 1986; Wanebo *et al.*, 1986). IL-2 is a glycoprotein of molecular weight approximately 15 kD. It is produced from lectin- or antigen-activated T cells, and has a number of functions, the most important being the stimulation of antigen-activated T cell proliferation (Smith, 1988).

The high affinity IL-2 receptor (IL-2R) is composed of two non-covalently linked subunits with molecular weights of 55 kD and 75 kD. Each is able to bind IL-2 with low affinity, but the complex allows binding to IL-2 to occur rapidly and dissociate very slowly. Signal transduction occurs solely via the 75 kD molecule, whereas the 55 kD molecule appears to act by aiding IL-2 binding (Kelly *et al.*, 1990). The IL-2R is the protein that mediates the action of IL-2. Normal resting B and T cells do not normally display significant numbers of these receptors on the cell surface (Robb *et al.*, 1981; Mizel, 1989). However, when such cells are stimulated by a challenge to the immune system, expression of IL-2R changes in two ways: some molecules of IL-2R are expressed on the plasma membrane and a form of IL-2R protein is released by the activated cells.

Recently serum IL-2R levels have been found to predict prognosis in patients with malignant disease. Lauria *et al.* (1992) found that patients with low levels of serum IL-2R at the time of diagnosis of Hairy-Cell Leukaemia (HCL) have a better chance of achieving a good clinical response while Fierro *et al.* (1992) estimated soluble IL-2R in 227 melanoma patients and found values in all stages significantly higher than in normal controls. Moreover these values correlated with disease progression. To date IL-2R has been evaluated extensively in haematological malignancies, but seldom in ovarian cancer. Kikuchi *et al.* (1988) studied IL-2 production by peripheral blood lymphocytes in advanced ovarian cancer during the course of combination chemotherapy. IL-2 levels were depressed but after addition of cimetidine, IL-2 production was restored. They found no difference between IL-2R expression in malignant compared to benign ovarian tumours. In the present study we compare serum IL-2R levels in patients with malignant ovarian tumours, normal ovaries and benign ovarian tumours.

Correspondence: O.J. Owens, Ward 4B, Department of Gynaecology, Stobhill General Hospital, Balornock Rd., Glasgow G21 3UW, UK.

Received 4 June 1992; an in revised form 8 March 1993.

### Materials and methods

### *Patient selection and serum collection*

Patients were recruited prospectively on the basis of a pre-operative clinical diagnosis of either malignant or benign ovarian tumour. All tumours were staged in accordance with FIGO classification (Shepherd, 1989) and subsequently classified in accordance with Serov *et al.* (1973) by a single pathologist. Age matched controls were identified pre-operatively in patients undergoing hysterectomy for benign conditions (usually menorrhagia) and their ovaries were all normal macroscopically. Ethical approval was obtained by the local ethical committee.

Ten ml of blood was taken pre-operatively or after clinical examination in the controls. Blood was centrifuged at 800 *g* for 10 min and serum was stored at − 20°C until required for the IL-2R assay.

### *IL-2 receptor assay*

This was measured using a sandwich enzyme immunoassay kit (Laboratory Impex Ltd). The detection limit of the assay is $50\,u\,ml^{-1}$ and the intra and interassay co-efficient of precision are 3.4% and 5.6% respectively.

### *Statistical analysis*

Wilcoxon and Mann-Whitney non parametric testing and Spearmans and Kendals regression analysis were used (Kmietowicz & Yannoulis, 1976).

### Results

Seventy-eight patients were available for analysis and comprised the following groups which are illustrated in Tables Ia and Ib: 30 patients with primary malignant ovarian tumours, five non ovarian cancer patients and one patient undergoing second look laparatomy (denoted by 'other' in Table Ia and Figure 1), 11 benign ovarian tumours, three uterine fibroids and 28 aged matched controls. For convenience the second look laparotomy is included in the 'other' group as this patient had received chemotherapy and all her biopsies were benign.

The 30 malignant ovarian tumours were subdivided with regard to stage and degree of differentiation as illustrated in Table Ia.

Figure 1 illustrates the distribution of the IL-2R levels. In the malignant ovarian tumour group the IL-2 levels ranged from 402 to 4,495 units $ml^{-1}$ ($U ml^{-1}$) with a median value of 1,267 $U ml^{-1}$. In the patients with benign ovarian tumours values ranged from 323–1,534 $U ml^{-1}$ with a median of 545 $U ml^{-1}$ while values in the control patients ranged from 309–1,177 $U ml^{-1}$ with a median of 567 $U ml^{-1}$. IL-2R levels were significantly elevated in the malignant group compared to the control group ($P<0.0001$), and the malignant group compared to the benign group ($P<0.0002$). There was no significant difference between the control group and benign ovarian tumour group.

There was no significant difference in IL-2R levels depending on stage and degree of differentiation in the malignant ovarian tumour group using Spearmans and Kendals regression analysis.

### Discussion

Kikuchi *et al.* (1988) found no difference in IL-2R levels between patients with benign tumours and those with ovarian carcinoma, but studied IL-2R expression in peripheral blood lymphocytes rather than serum as in this paper. This group also observed no change in IL-2R following chemotherapy and suggested that this may occur due to a decrease in IL-2 production and concomitant induction of IL-2R expression. Possibly the continued presence of tumour antigens could also explain this (Millis & Paetkau, 1980).

In contrast the results of Rovelli *et al.* (1988) agree with ours. In their study serum IL-2R levels were significantly higher in patients with malignant disease (mostly of breast and lung) than in normal subjects. Patients with metastatic solid tumours showed significantly higher mean levels than those with malignancy but without metastases, and similar to levels observed in the lymphoma patients. Interestingly, Lotze *et al.* (1987) found high levels of IL-2R positive lymphocytes in peripheral blood of patients with malignant tumours receiving recombinant IL-2.

This study has shown that IL-2R levels are significantly raised in patients with malignant ovarian tumours relative to normal controls. As this soluble IL-2R can bind interleukin-2, it may have an immunoregulatory role by competing with cellular IL-2R for the ligand and therefore down regulating the immune response. We found no correlation between IL-2R levels and disease staging or differentiation, or tumour bulk.

It was surprising to find that the IL-2R levels in the fibroid group were considerably higher than expected when compared to both controls and the benign ovarian tumour group. This could partly be explained by the fact that leiomyoma cells contain a stress responsive protein (SRP27) and also oestrogen and progesterone receptors which together have immunological properties similar to cancer cell lines (Navarro *et al.*, 1989). Fibroids have also been shown to produce erythropoietin suggesting that they have some immunological role. Further measurement of IL-2R levels in

**Table Ia** Epidemological data of patients

| *Groups* | *Age* | *Histological type* | *Stage* | *Differentiation* | *IL-2R* $(U\,ml)^{-1}$ |
|---|---|---|---|---|---|
| *Malignant ovarian tumours* | | | | | |
| | 69 | Serous | 3 | WD | 4495 |
| | 66 | Serous | 4 | WD | 853 |
| | 62 | Serous | 3 | WD | 553 |
| | 79 | Serous | 3 | MD | 1403 |
| | 78 | Serous | 3 | MD | 3711 |
| | 68 | Serous | 4 | MD | 2592 |
| | 54 | Serous | 3 | MD | 1964 |
| | 47 | Serous | 3 | MD | 3524 |
| | 54 | Serous | 3 | MD | 718 |
| | 68 | Serous | 1C | MD | 1045 |
| | 46 | Serous | 1A | MD | 800 |
| | 67 | Serous | 2A | PD | 1806 |
| | 79 | Serous | 4 | PD | 842 |
| | 60 | Serous | 1C | PD | 1226 |
| | 84 | Serous | 4 | Unstated | 1629 |
| | 53 | Clear cell | 2B | WD | 402 |
| | 61 | Clear cell | 2B | MD | 3076 |
| | 62 | Clear cell | 4 | MD | 778 |
| | 83 | Mucinous | 3 | MD | 2255 |
| | 54 | Mucinous | 4 | MD | 679 |
| | 79 | Endometrioid | 1C | WD | 486 |
| | 57 | Endometrioid | 3 | PD | 2514 |
| | 54 | Endometrioid | 3 | PD | 1666 |
| | 58 | Undifferentiated | 4 | | 768 |
| | 71 | Undifferentiated | 3 | | 1647 |
| | 67 | Undifferentiated | 4 | | 788 |
| | 69 | Unclassified | 3 | | 1730 |
| | 64 | Unclassified | 3 | | 1309 |
| | 74 | Mixed mesodermal | 3 | | 594 |
| | 28 | Endodermal sinus | 2B | | 959 |
| | 60 | Negative 2nd look laparotomy | | | 910 |
| *Non ovarian tumours* | | | | | |
| | 69 | Spindle cell low grade | | | 1348 |
| | 75 | Bladder tumour | | | 375 |
| | 70 | Caecal cancer | | | 751 |
| | 47 | Metastatic breast | | | 333 |
| | 45 | Fallopian tubal cancer | | | 912 |

Abbreviations: WD: Well differentiated. MD: Moderately differentiated. PD: Poorly differentiated.

**Table Ib** Epidemiological data of patients

| *Groups* | *Age* | *Histological type* | *IL-2R (U ml)*$^{-1}$ |
|---|---|---|---|
| Benign ovarian tumours | 48 | Teratoma | 637 |
| | 76 | Serous cystadenoma | 323 |
| | 74 | Serous cystadenoma | 404 |
| | 35 | Serous cystadenoma | 449 |
| | 51 | Mucinous cystadenoma | 638 |
| | 71 | Endometrioid borderline malignant | 570 |
| | 65 | Fibroma | 428 |
| | 69 | Fibroma | 696 |
| | 63 | Fibroma | 545 |
| | 56 | Simple cyst | 1534 |
| | 62 | Simple cyst | 413 |
| Fibroids | 84 | | 2577 |
| | 59 | | 2194 |
| | 52 | | 946 |
| Controls | 89 | | 1083 |
| | 84 | | 738 |
| | 81 | | 1067 |
| | 76 | | 503 |
| | 72 | | 329 |
| | 72 | | 602 |
| | 68 | | 532 |
| | 68 | | 469 |
| | 66 | | 849 |
| | 62 | | 608 |
| | 58 | | 836 |
| | 53 | | 911 |
| | 52 | | 824 |
| | 50 | | 416 |
| | 48 | | 309 |
| | 45 | | 442 |
| | 42 | | 465 |
| | 41 | | 467 |
| | 40 | | 400 |
| | 38 | | 476 |
| | 35 | | 362 |
| | 35 | | 515 |
| | 34 | | 715 |
| | 30 | | 1177 |
| | 29 | | 880 |
| | 27 | | 375 |
| | 21 | | 775 |
| | 20 | | 982 |

**Figure 1** Distribution of IL-2R values in U ml$^{-1}$ for the five different groups. The median values are shown by the bars.

patients with fibroids would be required to clarify this point.

We will assess at a later date whether an incidental measure of IL-2R pre-operatively has any bearing on long term prognosis. As has been suggested by others (Waldmann *et al.*, 1992), patients with malignant disease who demonstrate elevated IL-2R, may benefit therapeutically from IL-2. We are currently assessing whether patients with ovarian cancer have any alteration in thier IL-2R levels during chemotherapy or over the course of their disease.

This research was carried out with financial assistance from the Glasgow Royal Infirmary Gynaecology Oncology Fund.

### References

FIERRO, M.T., LISA, F., NOVELLI, M., BERTERO, M. & BERNENGO, M.G. (1992). Soluble interleukin-2 receptor, CD4 and CD8 levels in melanoma: a longititudinal study. *Dermatologica*, **184**, 182-189.

KELLY, S.A., MALIK, S. & BALKWILL, F.R. (1990). Cytokine therapy. In: *Ovarian Cancer, Biological and Therapeutic Challenges*. Sharp, F. Mason, W.P. & Leake, R.E. (eds), Chapman and Hall Medical: London. pp. 337-363.

KIKUCKI, Y., KIZAWA, I., OOMORI, K., IWANO, I., KITA, T., MIYAUCHI, M. & KATO, K. (1988). Effects of cimetidine on interleukin-2 production by peripheral blood lymphocytes in advanced ovarian carcinoma. *Eur. J. Cancer Clin. Oncol.*, **24**, 1185-1190.

KMIETOWICZ, Z.W. & YANNOULIS, Y. (1976). *Mathematical Statistical and Financial Tables for the Social Sciences*. Longman: London. 33-36.

LAURIA, F., RONDELLI, D., RASPADORI, D., ZINZANI, P.L., BENFENATI, D., PILERI, S., SABATTINI, E. & TURA, S. (1992). Serum soluble interleukin-2 receptor levels in hairy cell leukaemia: correlation with clinical and haematological parameters and with Alpha-interferon treatment. *Leuk-Lymphoma*, **7**, 103-107.

LOTZE, M.T., CUSTER, M.C., SHARROW, S.O., RUBIN, L.A., NELSON, D.L. & ROSENBERG, S.A. (1987). *In vivo* administration of purified human interleukin-2 to patients with cancer: development of interleukin-2 receptor positive cells and circulating soluble interleukin-2 receptors following interleukin-2 administration. *Cancer Res.*, **47**, 2188-2195.

MANTOVANI, G., COIANA, A., COSSU, F., FLORIS, C., PROTO, E., MACCIO, A., PISANO, G., TAGLIERI, G., PUXEDDU, G. & DEL GIACCO, G.S. (1986). Peripheral blood lymphocytes response to exogenous interleukin 2 by PHA-prestimulated and non PHA-prestimulated cells in patients with cancer. *Tumori*, **72**, 375-382.

MILLIS, G.B. & PAETKAU, V. (1980). Generation of cytotoxic lymphocytes to syngeneic tumor by using co-stimulator (interleukin 2). *J. Immunol.*, **125**, 1897-1903.

MIZEL, S.B. (1989). The interleukins. *FASEB J.*, **3**, 2379-2388.

NAKAYAMA, E., ASANO, S., TAKUWA, N., YOKOTA, J. & MIWA, S. (1983). Decreased TCGF activity in the culture medium of PHA stimulated peripheral mononuclear cells from patients with metastatic cancer. *Clin. Exp. Immunol.*, **51**, 511-516.

NAVARRO, D., CABRERA, J.J., FALCON, O., JIMENEZ, P., RUIZ, A., CHIRINO, R., LOPEZ, A., RIVERO, J.F., DIAZ-CHICO, J.C. & DIAZ-CHICO, B.N. (1989). Monoclonal antibody characterization of progesterone receptors, estrogen receptors and the stress-responsive protein of 27 kDa (SRP27) in human uterine leiomyoma. *J. Steroid Biochem.*, **34**, 491-498.

ROBB, R.J., MUCK, A. & SMITH, K.A. (1981). T-cell growth factor receptors. Quantitation, specificity, and biological relevance. *J. Exp. Med.*, **154**, 1455-1458.

ROVELLI, F., LISSONI, P., CRISPINO, S., BARNI, S., FUMAGALLI, G., PAOLOROSSI, F. & TANCINI, G. (1988). Increased level of soluble interleukin-2 receptor in advanced solid tumours: A preliminary study. *Tumori.*, **74**, 633-637.

SEROV, S.F., SCULLY, R.E. & SOBIN, L.H. (1973). *International Classification of Tumours*. No. 9. Histological typing of ovarian tumours. World Health Organization: Geneva.

SHEPHERD, J.H. (1989). Revised FIGO staging for gynaecological cancer. *Br. J. Obstet. Gynaec.*, **96**, 889-892.

SMITH, K.A. (1988). Interleukin-2: inception, impact and implications. *Science*, **240**, 1169-1176.

WALDMANN, T.A., PASTAN, I.H., GANSOW, O.A. & JUNGHANS, R.P. (1992). The multichain interleukin-2 receptor: a target for immunotherapy. *Ann Intern Med.*, **116**, 148-160.

WANEBO, H.J., PACE, R., HARGETT, S., KATZ, D. & SANDO, J. (1986). Production of and response to interleukin-2 in peripheral blood lymphocytes of cancer patients. *Cancer*, **57**, 656-662.